# Prevention of Bradycardia during Spinal Anesthesia under Dexmedetomidine Sedation in Older Adults

**DOI:** 10.3390/jcm11216349

**Published:** 2022-10-27

**Authors:** Seyoon Kang, Yun Jeong Chae, Sun Kyung Park, Taek Geun Kim, Han Bum Joe

**Affiliations:** 1 Department of Anesthesiology and Pain Medicine, Ajou University School of Medicine, Suwon 16499, Korea; 2 Department of Anesthesiology and Pain Medicine, Jeju National College of Medicine, Jeju 63241, Korea

**Keywords:** dexmedetomidine, spinal anesthesia, bradycardia, geriatric, pretreatment

## Abstract

Older adults exhibit reduced physiological responses to beta-adrenergic stimulation and parasympathetic inhibition. This study aimed to investigate the effect of reducing the incidence of bradycardia in the atropine and ephedrine pretreatment group compared to the control group in older adults who received spinal anesthesia with intravenous dexmedetomidine. Overall, 102 older adults aged over 65 years were randomly divided into three groups, and saline (control group), atropine at 0.5 mg (atropine group), and ephedrine at 8 mg (ephedrine group) were administered intravenously to each group as pretreatment. Immediately after spinal anesthesia, dexmedetomidine loading and study drug injections were commenced. The primary outcome was the incidence of bradycardia (<50 beats per min) within 60 min following dexmedetomidine loading. The incidence of bradycardia requiring atropine treatment was significantly higher in the control group than in the atropine and ephedrine groups (27.3% vs. 6.1% and 8.8%, respectively; *p* = 0.035), and no difference was noted between the atropine and ephedrine groups. Therefore, if ephedrine or atropine is selected and used according to the patient’s condition and clinical situation, it may be helpful in preventing bradycardia during spinal anesthesia using dexmedetomidine in older patients.

## 1. Introduction

Adequate sedation during regional anesthesia is often required to relieve patient’s anxiety, clear noxious memories, increase comfort during lengthy surgeries (especially in uncomfortable postures), and make it easier for the patient to accept regional anesthesia [1]. Among several sedatives, propofol and dexmedetomidine are the most common [2]. Although the onset of action of dexmedetomidine is somewhat slower than that of propofol, dexmedetomidine has several advantageous properties such as absent or insignificant respiratory depression even at high plasma concentration, stable hemodynamic profile, improved quality and duration of sensory and motor block of spinal anesthesia, and improved postoperative analgesia [3,4,5,6]. In addition, it has been reported to be effective in reducing postoperative delirium in older adults [7].

However, the use of dexmedetomidine during spinal anesthesia has been associated with an increased incidence of bradycardia [6,8,9,10]. In previous studies of pretreatment with intravenous atropine and intramuscular ephedrine for the prevention of bradycardia in relatively young patients, both pretreatments were reported to be effective [11,12]. In contrast, responses of the geriatric population to pretreatments may not be the same because an age-associated decline in cardiovascular performance leads to a reduced response to beta-adrenergic stimulation and parasympathetic inhibition [13,14].

Therefore, this study aimed to investigate the effect of reducing the incidence of bradycardia in the atropine and ephedrine pretreatment groups when compared with that the control group in older adults who received spinal anesthesia with intravenous dexmedetomidine.

## 2. Materials and Methods

This was a single-center, prospective, double-blind, randomized controlled trial with three parallel groups performed at Ajou University Hospital (Suwon, South Korea) from June 2019 to September 2020. The trial was approved by the institutional review board of Ajou University Hospital (AJIRB-MED-THE-18-343) and was registered at ClinicalTrials.gov (NCT no. 03984526). Written informed consent was obtained from all the patients.

In total, 102 older adults (aged > 65 years) with an American Society of Anesthesiologists (ASA) physical status of I or II undergoing knee arthroplasty under spinal anesthesia were enrolled in this study. The following patients were excluded from this study: those with contraindications to spinal anesthesia; those with arrhythmia or conduction abnormality, uncontrolled preoperative hypertension (systolic blood pressure > 160 mmHg or diastolic pressure > 100 mmHg), and unstable angina or cardiomyopathy; and those taking β-adrenergic blockers, anticholinergics, or any other drugs that may alter the normal response to the study drugs. Additionally, patients were excluded if they failed spinal anesthesia or had a sensory block level lower than T10 or higher than T7.

Patients were randomly allocated in a 1:1:1 ratio to one of the three (control, atropine, and ephedrine) groups, with 34 patients per group, using the Excel “Random” function (Microsoft Office 2010, Microsoft Corporation, Redmond, WA, USA). The allocation process was conducted by a colleague not involved in this study. The randomization results were concealed within serially numbered opaque envelopes. Before the patient was taken to the operating room, the numbered envelope was opened, and a nurse not participating in this study prepared 2 mL of the study drug assigned in a 5-mL syringe. All parties involved, including the patients, anesthesiologists, and investigators collecting the data, were unaware of the study drugs or the patient group assignment.

All patients underwent the same anesthetic protocol. Patients were not administered any premedication. Before spinal anesthesia, each patient was administered 5 mL/kg (based on ideal body weight) of crystalloid for preloading. Intraoperative monitoring included electrocardiography, noninvasive blood pressure measurement, pulse oximetry, and ADMS^TM^ (Anesthetic Depth Monitor for Sedation, Unimedics CO., LTD., Seoul, Korea). Spinal anesthesia was performed using a 25-gauge Quincke needle between L4 and L5 or L3 and L4 with the patient in the lateral decubitus position using the paramedian approach. The amount of hyperbaric bupivacaine (Marcaine^®^ Spinal 0.5% Heavy, AstraZeneca, Cambridge, UK) was determined by an anesthesiologist with more than 10 years of experience. This amount was determined according to the height, weight, and age of the patient and in consideration of the target sensory level of anesthesia at T10. After administration of the intrathecal drug, the patient’s position was immediately changed to supine. The sensory block level was assessed by the pinprick test using a 24-gauge hypodermic needle and recorded 10 min after the patient was placed in the supine position.

Immediately after spinal anesthesia, dexmedetomidine (Precedex^TM^, Pfizer, New York, NY, USA) was infused at a loading dose of 0.6 μg/kg for 10 min, followed by an infusion at 0.5 μg/kg/h. Simultaneously with the initiation of the dexmedetomidine loading, patients in the control group received an intravenous normal saline bolus, whereas patients in the atropine group received an intravenous bolus of 0.5 mg atropine and patients in the ephedrine group an intravenous bolus of 8 mg ephedrine. Sedation status was measured every 10 min using the Ramsay sedation scale (1 = anxious and agitated; 2 = cooperative and tranquil; 3 = responds to commands only; 4 = brisk response to light glabellar tap or auditory stimulus; 5 = sluggish response to a light glabellar tap or auditory stimulus; and 6 = no response to a light glabellar tap or auditory stimulus) [15]. A Ramsay sedation score of 3 or higher was considered an adequate level of sedation. If a patient did not achieve an adequate level of sedation 20 min after the start of dexmedetomidine loading, midazolam bolus at 1 mg was administered intravenously up to three times.

Systolic/mean/diastolic blood pressure (SBP/MBP/DBP), heart rate (HR), Ramsay sedation score, and uCON index of ADMS, which is identical to the qCON index (Quantium Medical, Barcelona, Spain) [16] were recorded before spinal anesthesia (baseline); at the start of dexmedetomidine loading (initial); and at 1, 5, 10, 15, 20, 30, 40, 50, and 60 min after the loading of dexmedetomidine. At the end of the surgery, dexmedetomidine infusion was stopped, and patients were transferred to the postoperative care unit. Bradycardia was defined as HR < 50 beats per min and was treated with intravenous atropine (0.5 mg). Hypotension was defined as either an SBP reduction of >30% compared with baseline SBP or SBP < 90 mmHg; it was treated with 100 mL of intravenous crystalloid fluid replacement and intravenous ephedrine (4 mg). If hypertension occurred (SBP increased by 30% or more than baseline SBP or SBP of 200 mmHg or more), 250 μg of nicardipine was injected intravenously [11].

The primary outcome was the incidence of bradycardia within 60 min after dexmedetomidine loading. The secondary outcomes were the incidences of hypertension and hypotension, changes in hemodynamic data (SBP, MBP, DBP, and HR), and the number of midazolam boluses administered.

In a similar study in which spinal anesthesia was performed under dexmedetomidine sedation in older adults, the incidence of bradycardia was reported as 40% [10]. Based on the assumption that atropine or ephedrine pretreatment would reduce this incidence to within 10%, the sample size required in each group was 31, with a power of 0.8 and a level of significance of 0.05. The number of patients in each group was 34 in this study in consideration of the possibility of dropout.

We used the Statistical Package for the Social Sciences (version 11.0, SPSS Inc., Chicago, IL, USA) for statistical analyses. Data are presented as mean ± standard deviation, median (interquartile range), or the number of patients. Continuous variables were analyzed using one-way analysis of variance (ANOVA) or the Kruskal–Wallis test depending on normality test results. We used the Kolmogorov–Smirnov test for normality testing of the variable distribution. Categorical variables were analyzed using the chi-square test or Fisher’s exact test. Hemodynamic and uCON variables over time among the groups were analyzed using repeated-measures ANOVA and followed by the Bonferroni method as a post hoc multiple comparison. For cross-sectional comparison among groups, one-way ANOVA was used. A *p* value < 0.05 was considered to be statistically significant.

## 3. Results

**Figure 1:**
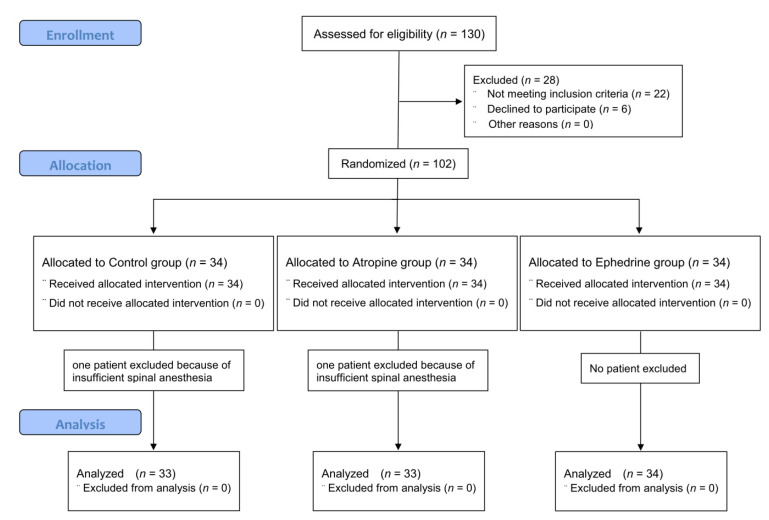
CONSORT flow diagram of recruitment and assessment of study participants.

In total, 130 older adults were screened, of whom 6 patients refused participation, and 22 patients were excluded because they did not meet the inclusion criteria. The participant flow diagram for the study is presented in Figure 1. Two patients (one in the control group and one in the atropine group) underwent general anesthesia due to failure of spinal anesthesia and were excluded from the analysis. Therefore, data from 100 patients were included in the analysis.

Patient characteristics and clinical data are presented in Table 1. No significant difference was noted among the three groups in sex, age, height, weight, preoperative hemodynamics, and uCON values. Moreover, no significant difference was found among the groups in anesthesia and operation time.

The incidence rates of rescue treatment for hemodynamics and sedation are presented in Table 2. The incidence of bradycardia requiring atropine treatment was significantly higher in the control group than in the atropine and ephedrine groups (27.3% vs. 6.1% and 8.8%, respectively; *p* = 0.035). There was no difference between the atropine and ephedrine groups. Rescue atropine administration time was within 20 min of starting dexmedetomidine in the control group; 5, 20, and 26 min in the atropine group; and 15, 30, and 52 min in the ephedrine group. Contrastingly, the incidence of hypotension requiring ephedrine treatment was not significantly different among groups (0% in the control group vs. 3% in the atropine group vs. 0% in the ephedrine group; *p* = 0.666). Furthermore, the incidence of hypertension requiring nicardipine treatment was not significantly different among groups (9.1% in the control group vs. 15.2% in the atropine group vs. 5.9% in the ephedrine group; *p* = 0.417). All these hypertensive events occurred 5–10 min after dexmedetomidine loading. Ramsay sedation scores at 10, 15, and 20 min after dexmedetomidine administration were 2.5 (2.0–3.0), 3.0 (2.0–4.0), and 4.0 (3.0–4.0), respectively. However, more than half the patients required additional administration of midazolam. The incidence of patients requiring midazolam to meet satisfactory sedation with dexmedetomidine was not significantly different among the groups (69.6% in the control group vs. 63.6% in the atropine group vs. 52.9% in the ephedrine group; *p* = 0.668).

**Figure 2:**
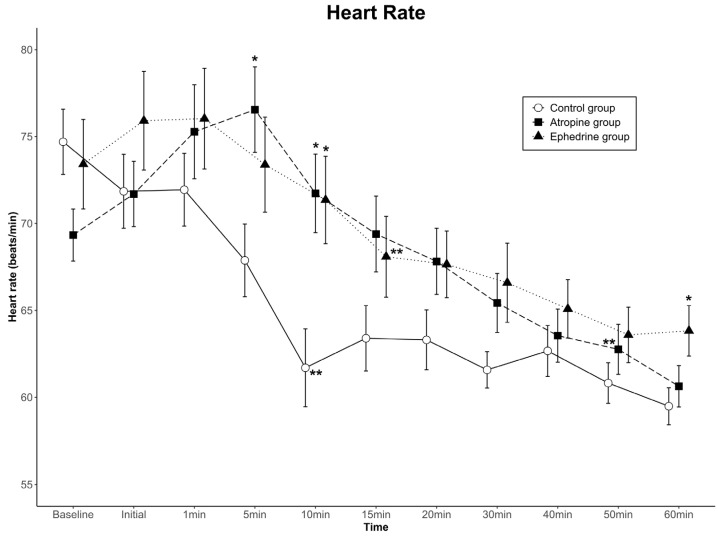
Heart rate (HR) values for the three groups. Data are presented as mean ± standard error. A significant time-by-group interaction was observed among the groups (*p* < 0.001). The HRs at 5 and 10 min in the atropine group were higher than those in the control group, and the HRs at 10 and 60 min in the ephedrine group were higher than those in the control group (* *p* < 0.05 compared to the control group in the same period by one-way ANOVA). The HR was maintained at lower than the baseline HR of each group from 10 min in the control group, 15 min in the ephedrine group, and 50 min in the atropine group (** *p* < 0.05 compared to baseline within the group by Bonferroni method as a post hoc multiple comparison after repeated-measures ANOVA). Baseline, baseline value before spinal anesthesia: Initial, immediately before the administration of dexmedetomidine; 1–60 min, 1–60 min after the administration of dexmedetomidine.

The data for HR are shown in Figure 2. Comparing the differences among groups in the HR change at successive time points, no statistically significant difference was found in HR over the 11 time points (group effect, *p* = 0.222). In contrast, a significant difference was noted in the time-by-group interaction (*p* < 0.001). In the control group, the HR at 5 min was significantly lower than that in the atropine group; at 10 min, it was significantly lower than those in the atropine and ephedrine groups; and at 60 min, it was significantly lower than that in the ephedrine group. Conversely, the change in HR with time showed a significant difference (time effect, *p* < 0.001). When the HR at each time point was compared with the baseline HR within the group, the HR values were lower than the baseline values from 10 min in the control group and 15 min in the ephedrine group after dexmedetomidine loading. Contrarily, the HR values of the atropine group were relatively well maintained and became lower than the baseline value from 50 min after dexmedetomidine loading. Overall, the HR was clinically stable in all groups.

**Figure 3:**
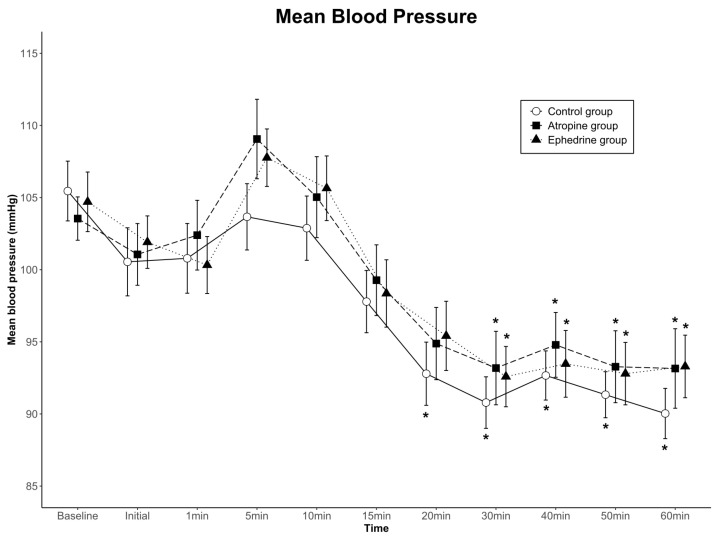
Mean arterial blood pressure values for the three groups. Data are presented as mean ± standard error. There were no significant differences among the groups, and no significant interactions with time-by-group. * *p* < 0.05 compared to baseline within group by one-way ANOVA. Baseline, baseline value before spinal anesthesia; Initial, immediately before the administration of dexmedetomidine; 1–60 min, 1–60 min after the administration of dexmedetomidine.

The data for mean arterial blood pressure are shown in Figure 3. Although blood pressure for each of the three groups changed over time (*p* < 0.001), no significant differences were found among the groups (group effect; *p* = 0.666 in SBP, *p* = 0.738 in MBP, *p* = 0.810 in DBP). No significant interactions were observed with time-by-group (*p* = 0.383 in SBP, *p* = 0.772 in MBP, *p* = 0.386 in DBP). Overall, blood pressure was clinically stable in all groups.

## 4. Discussion

In this randomized controlled trial that assessed the bradycardia-preventive effect of atropine and ephedrine pretreatment administered simultaneously with the start of dexmedetomidine infusion, immediately after spinal anesthesia in older adults, we found that atropine and ephedrine reduced the incidence of bradycardia by a quarter and one-third when compared with that in the control group, respectively. No difference was noted between atropine and ephedrine pretreatment. This study shows that even in older adults who exhibit reduced physiological responses to beta-adrenergic stimulation and parasympathetic inhibition, atropine or ephedrine pretreatment is effective in preventing bradycardia in spinal anesthesia under sedation with dexmedetomidine. In addition, spinal anesthesia with dexmedetomidine did not induce hypotension in older adults except in one patient. Hypertension was observed more frequently than hypotension. All the hypertensive events occurred 5–10 min after dexmedetomidine administration.

Bradycardia is known to occur in approximately 13% of spinal anesthesia patients, with a higher incidence in those with baseline HR < 60 beats per min, those with ASA physical status I, those who use beta-adrenergic blockers, and those with block height T5 or higher [17]. Bradycardia due to the use of dexmedetomidine in spinal anesthesia occurs in approximately 13–30% of patients, depending on the difference in the loading dose of dexmedetomidine [9,11]. Bradycardia caused by dexmedetomidine is effectively prevented by pretreatment with atropine or ephedrine in young patients [11,12]. However, whether the same results are obtained in older adults should be confirmed. With age, cardiac output response to beta-adrenergic stimulation decreases as a result of the decline in the sympathetic control of the heart [14]. In addition, decreased cardiac vagal control with aging, including decreased M2 muscarinic receptor density and function [18] and impaired cardiac acetylcholine release, occurs in response to stimulation [19], resulting in decreased cardiovascular response to atropine-induced parasympathetic inhibition with aging [13]. Therefore, in the pretreatment for the prevention of bradycardia caused by dexmedetomidine, the response to both sympathetic stimulation and parasympathetic inhibition in older adults may theoretically be decreased compared to that in younger patients [13]. Contrary to this theoretical possibility, in this study, dexmedetomidine-induced bradycardia was prevented in older adults with both ephedrine and atropine pretreatment, similar to that in younger patients [11]; therefore, the decreased responsiveness to sympathomimetic and anticholinergic agents in older adults was not evident in this study. In addition, atropine pretreatment, compared with ephedrine pretreatment, adequately maintained the patient’s HR for a relatively long duration. In the ephedrine group, the HR was lower than the baseline HR from 15 min after dexmedetomidine loading, whereas in the atropine group, the HR was lower than the baseline HR only 50 min after loading dexmedetomidine, indicating slightly longer bradycardia prevention in the atropine group. This difference is believed to be because ephedrine pretreatment has a shorter duration of action than atropine pretreatment in this study. Interestingly, however, despite this difference, the incidence of bradycardia requiring overall atropine rescue treatment was less in both pretreatment groups than in the control group. As seen in the control group, dexmedetomidine-induced bradycardia generally occurred within 20 min after the initiation of dexmedetomidine infusion. The reason why the two pretreatments showed comparable bradycardia prevention effects is presumably because ephedrine pretreatment, which has a short duration of action, prevented bradycardia during this period.

The use of loading doses of dexmedetomidine may cause a transient increase in blood pressure [20], which may be more severe when combined with pretreatment with atropine or ephedrine [21,22]. However, dexmedetomidine is considered an appropriate choice in spinal anesthesia because transient increase in blood pressure with dexmedetomidine may be effective in offsetting hypotension due to spinal anesthesia. In this context, this study using dexmedetomidine showed a very low incidence of hypotension, whereas previous studies on older adults reported hypotension in 27–69% of patients after spinal anesthesia [23,24].

When using a loading dose of dexmedetomidine, it is known that a high loading dose (1.0–2.0 µg/kg) showed a biphasic response with hypotension after a temporary increase in blood pressure, whereas a low loading dose (0.25–0.5 µg/kg) revealed a monophasic response with slow onset hypotension without an increase in blood pressure [20]. In addition, Ko et al. have reported that a dose of 0.5 µg/kg or more for the use of a dexmedetomidine loading dose during spinal anesthesia may cause hemodynamic instability and that the effective dose for adequate sedation was 0.86 µg/kg in 95% of patients receiving dexmedetomidine loading dose [25]. Therefore, the authors of this study attempted to obtain appropriate sedation and hemodynamic stability by selecting 0.5 µg/kg/h as the maintenance infusion rate after a dexmedetomidine loading dose of 0.6 µg/kg for 10 min. In this protocol, additional doses of intravenous midazolam were required to achieve adequate sedation 20 min after the start of dexmedetomidine loading: 1 mg in 50 patients (50%), 2 mg in 10 patients (10%), and 3 mg in 3 patients (3%). In a study of the effects of propofol and midazolam sedation on cognitive impairment, the only modifiable factor predicting cognitive impairment in individuals was administration of midazolam > 2 mg [26]. Therefore, it seems that our protocol can be used without concern for cognitive decline in most cases. However, the combined effects of dexmedetomidine and midazolam are unclear, and in some cases, the disadvantages of midazolam may offset the advantages of dexmedetomidine on cognitive function. From a hemodynamic point of view, despite the reduction in the loading dose of dexmedetomidine, in some patients, a transient increase in blood pressure was observed about 5 min after loading dexmedetomidine regardless of the group, and the incidence of bradycardia was not small in the control group. Thus, when dexmedetomidine is used in spinal anesthesia in older adults, controlling the infusion rate of dexmedetomidine or co-administration with other drugs may be required for proper sedation and prevention of hypertension and bradycardia, which are topics of future study.

In this study, pretreatment with ephedrine or atropine did not increase the incidence of hypertension or tachycardia requiring treatment, compared with the control group, nor did hemodynamic and respiratory morbidity or mortality occur. Minor side effects such as nausea and dizziness due to ephedrine or atropine were not included in the study. In this study, the absence of major adverse events may be due to the combination of the hemodynamic offsetting effect of medications and spinal anesthesia. However, it may also be due to the small number of samples and meticulous monitoring and management of relatively healthy older patients aged approximately 70 years. With aging, older adults become increasingly susceptible to the hemodynamic effect of anesthetic agents and other cardiovascular stressors [27]. Older adults with or without medical complications require personalized hemodynamic management based on the individual personal baseline to reduce major postoperative complications [28]. Therefore, the results of our study should be applied with caution.

This study had some limitations. First, the dose selection of dexmedetomidine may differ depending on the user; thus, the incidence of bradycardia and the effect of pretreatment may vary depending on which dose is administered as a bolus and how much is administered as a maintenance dose. Second, in this study, when dexmedetomidine was used in older adults, the dose of dexmedetomidine was minimized as much as possible for hemodynamic stability. Therefore, midazolam was used when sedation was insufficient, which may have affected the research results. However, the hemodynamic effect of midazolam is a decrease in blood pressure due to a decrease in systemic vascular resistance. It has no effect on HR and is considered insignificant in the small doses used in this study. Third, since this study was conducted on patients who underwent knee arthroplasty, female patients were predominant due to the epidemiologic characteristics of knee arthroplasty in Korea [29]. Therefore, the data of this study should be interpreted in consideration of sex differences in pharmacokinetics and pharmacodynamics of dexmedetomidine. Fourth, the sample size was selected to examine whether the pretreatment group exhibited bradycardia prevention effects compared to that exhibited by the control group; this was not a study that could demonstrate the difference between the two pretreatment groups.

## 5. Conclusions

In conclusion, compared to the control group, ephedrine and atropine pretreatment prevented bradycardia when dexmedetomidine was used in spinal anesthesia in older adults, and no difference was found between the ephedrine and atropine groups. Therefore, if ephedrine or atropine is selected and used according to the patient’s condition and clinical situation, it may help prevent bradycardia during spinal anesthesia under dexmedetomidine sedation in older patients.

## Figures and Tables

**Table 1:** Patient characteristics and clinical data.

Variables	Control Group (*n* = 33)	Atropine Group (*n* = 33)	Ephedrine Group (*n* = 34)	*p*-Value
Sex (M/F)	3/30	3/30	6/28	0.46
Age (years)	72.0 (68.0–74.0)	71.0 (67.0–77.5)	73.0 (69.8–77.3)	0.38
Height (cm)	154.3 ± 5.0	155.6 ± 6.9	154.4 ± 7.8	0.67
Weight (kg)	61.5 ± 8.6	63.7 ± 8.0	61.7 ± 11.4	0.54
ASA class (I/II)	8/25	11/22	12/22	0.63
Medical history				
Hypertension (Y/N)	24/9	21/12	20/14	0.49
Diabetes mellitus (Y/N)	4/29	7/26	5/29	0.63
ECG (normal/abnormal)	20/13	24/9	25/9	0.45
Baseline HR	74.7 ± 10.8	69.3 ± 8.6	73.4 ± 15.0	0.16
Baseline SBP	155.0 (146.0–159.0)	149.0 (136.0–159.0)	150.0 (144.8–154.5)	0.22
Baseline MBP	105.5 ± 11.9	103.5 ± 8.6	104.7 ± 12.0	0.78
Baseline DBP	78.4 ± 9.1	78.6 ± 9.4	79.5 ± 10.9	0.89
Baseline uCON	97.0 (95.0–98.0)	97.0 (94.0–98.0)	97.0 (96.0–98.0)	0.83
Sensory block level, Thoracic	9.0 (7.5–10.0)	9.0 (8.0–10.0)	8.5 (7.8–10.0)	0.63
Anesthesia time (min)	170.0 (160.0–185.0)	175.0 (162.5–185.0)	175.0 (163.8–200.0)	0.20
Operation time (min)	100.0 (95.0–112.5)	110.0 (97.5–115.0)	105.0 (100.0–135.0)	0.07

Note: Values are presented as number, mean ± standard deviation or median (interquartile range). M, male; F, female; ASA, American Society of Anesthesiologists; Y, yes; N, no; ECG, electrocardiogram; HR, heart rate; SBP, systolic blood pressure; MBP, mean arterial blood pressure; DBP, diastolic blood pressure; min, minutes.

**Table 2:** Incidence rates of rescue treatment for hemodynamics and sedation.

Variables	Control Group (*n* = 33)	Atropine Group (*n* = 33)	Ephedrine Group (*n* = 34)	*p*-Value
Rescue Atropine (1/2)	8/1 (27.3)	1/1 (6.1)	3/0 (8.8)	0.035
Rescue Ephedrine (1/2)	0/0 (0.0)	1/0 (3.0)	0/0 (0.0)	0.66
Rescue Nicardipine (1/2)	3/0 (9.1)	5/0 (15.2)	2/0 (5.9)	0.42
Rescue Midazolam (1/2/3)	18/4/1 (69.7)	16/4/1 (63.6)	16/2/0 (52.9)	0.67

Note: Values are presented as the number of patients (% of patients required rescue treatment). (1/2/3) indicates the number of administrations of rescue medication.

## Data Availability

The data that support the findings of this study are available from the corresponding author upon reasonable request.
